# Tuberculosis contact tracing yield and associated factors in Uganda

**DOI:** 10.1186/s12890-022-01860-z

**Published:** 2022-02-16

**Authors:** Joseph Baruch Baluku, Martin Nabwana, Muttamba Winters, Felix Bongomin

**Affiliations:** 1 Makerere University Lung Institute, Kampala, Uganda; 2 Kiruddu National Referral Hospital, Kampala, Uganda; 3 Makerere University – Johns Hopkins University Research Collaboration, Kampala, Uganda; 4 Department Medical Microbiology and Immunology, Faculty of Medicine, Gulu University, Gulu, Uganda

**Keywords:** Contact tracing, Tuberculosis, Yield, Presumptive, Active, Uganda

## Abstract

**Background:**

The yield of tuberculosis (TB) contact tracing is historically low in Uganda. We determined factors associated with a positive contact tracing yield at an urban public TB clinic in Kampala, Uganda.

**Methods:**

We reviewed contact tracing registers of index TB cases registered between 2015 and 2020 at Kitebi Health Center, a primary level facility. Contacts who had symptoms of TB were designated as having presumptive TB. A contact investigation that yielded a new TB case was designated as a positive yield. We used logistic regression to determine factors associated with a positive yield of contact tracing.

**Results:**

Of 778 index TB cases, 455 (58.5%) had a contact investigation conducted. Index cases with a telephone contact in the unit TB register (adjusted odds ratio (aOR) 1.66, 95% CI 1.02–1.97, *p* = 0.036) were more likely to have a contact investigation conducted than those who did not. Of 1350 contacts, 105 (7.8%) had presumptive TB. Of these, 73 (69.5%) were further evaluated for active TB and 29 contacts had active TB. The contact tracing yield for active TB was therefore 2.1% (29/1,350). The odds of a positive yield increased tenfold with each additional presumptive contact evaluated for active TB (aOR 10.1, 95% CI 2.95–34.66, *p* < 0.001). Also, retreatment index TB cases were more likely to yield a positive contact (aOR 7.69 95% CI 2.08–25.00, *p* = 0.002) than to new cases.

**Conclusion:**

TB contact tracing should aim to evaluate all contacts with presumptive TB and contacts of retreatment cases to maximise the yield of contact tracing.

**Supplementary Information:**

The online version contains supplementary material available at 10.1186/s12890-022-01860-z.

## Introduction

The global burden of tuberculosis (TB) is falling at a slow rate. There was a 9% decline in the TB incidence between 2015 and 2019 against a 20% target that is required to achieve the 2020 milestones of the End TB strategy [1]. In the 2018 United Nations High-Level Meeting on TB, member states committed to providing TB treatment to at least 40 million people with TB between 2018 and 2022 [2]. However, many people with TB are not diagnosed and this target may not be achieved. An estimated 2.9 million people who developed TB in 2019 were not notified either because they did not receive a TB diagnosis or were not reported to national TB programs [2]. Strategies to increase the detection of TB are urgently needed. Active case finding strategies such as screening for TB through outreaches, health promotion through mass media and locally organised events to encourage uptake of screening services and mobile TB screening clinics increase case detection [3]. This is observed in populations at risk of TB and the general population [4].

Contact tracing is the systematic evaluation of household and close social contacts of an index TB case to identify additional TB cases [5]. In addition to identifying additional TB cases, contact tracing is associated with treatment success among index TB cases [6, 7]. The World Health Organisation (WHO) recommends contact tracing among household and close contacts of an index TB case who has any of sputum-positive pulmonary TB, drug resistant TB, HIV co-infection or is a child aged < 5 years [5]. Unfortunately, the yield of a new TB case is low during contact investigations among these groups of individuals. The prevalence of active TB among contacts of index TB cases is only 3% in low- and middle-income countries [8]. Additionally, the median number needed to screen to identify a single active TB case is very varied; from 14 household contacts in high TB incidence countries to 104 community contacts in low incidence countries [9]. Strategies to increase the yield of TB contact tracing are needed. The characteristics of the index TB case and their contacts are important in determining the initiation and scope of contact tracing [10]. Identifying factors associated with a positive contact tracing yield would help programs fine-tune contact tracing strategies to increase the positive yield and cost-effectiveness of contact investigations, particularly in low-income settings.

Recently, Uganda was reclassified as a TB and TB/HIV high-burdened country by WHO [11]. The yield of contact tracing in Uganda is historically low. Only about 1.7–5.3% of contacts of an index TB case who undergoes contact tracing turn out to have active TB [12–14]. Consequently, contact tracing may not be cost effective due to the low positivity yield [14]. In this study, we determined factors associated with a positive contact tracing yield among index TB cases at an urban TB clinic in Kampala, Uganda.

## Material and methods

### Study population, design and setting

As part of a larger study [6], we conducted a retrospective review of unit TB and contact tracing registers at Kitebi Health Center (KHC), an urban public health facility in Kampala, the capital city of central Uganda. KHC is a primary level facility where people seek general health services. The objective of the primary study was to determine the effect of contact tracing on TB treatment outcomes. Eligible index TB cases in the primary study were individuals (including children and extrapulmonary TB) who received treatment from KHC between 2015 and 2020 and had a documented treatment outcome. Treatment outcomes were cure, death, loss-to-follow up and treatment failure. Cases with drug resistant TB and those transferred out to another facility were excluded. The TB clinic at KHC offers outpatient services to a largely urban poor population from Kampala and Wakiso districts of Uganda. Index and presumptive TB cases were diagnosed with pulmonary bacteriologically confirmed TB by way of an MTB Xpert/RIF assay or sputum microscopy by standard national guidelines [15]. Cases were diagnosed with pulmonary clinically diagnosed TB if they had suggestive symptoms, chest imaging, and the attending clinician decided to initiate a full course of anti-tuberculous medication [16]. New cases were individuals who had never been treated for TB or received TB therapy for < 1 month while retreatment cases had received at least one month of TB therapy in the past before the current diagnosis [16].

Cases with bacteriologically confirmed TB, TB/HIV co-infected cases and children < 5 years of age were recommended to have contact tracing done [17]. Upon TB diagnosis, health workers elicited household and social contacts of index cases through a face-to-face interview conducted at the health facility. Thereafter, health workers contacted the contacts via telephone (or asked the index case to relay information to the contact) to schedule a visit at the home or workplace of the contact or asked the contact to come to the health facility for evaluation. Contacts were screened for TB symptoms: cough for ≥ 2 weeks or any duration for people with HIV, persistent fevers, weight loss or anorexia and night sweats; using the intensified case finding form [14] (Additional file 1). Any contact with any of the symptoms above was designated as having presumptive TB. Presumptive TB cases identified through a home or workplace visit were referred to KHC for further evaluation for TB. A contact diagnosed with bacteriologically confirmed or clinically diagnosed TB was considered to be a “positive yield”.

### Study tools and measurements

Data were collected from the registers using a data abstraction form. From the unit register, we collected characteristics of the index cases. Specifically, sociodemographic data, HIV status, type of case (new or retreatment (relapse, failure, return after loss to follow up)), and TB class (pulmonary bacteriologically confirmed, pulmonary clinically diagnosed and extra-pulmonary TB) were collected. From the contact tracing register, we extracted data on number of times contact tracing was conducted for a given index case, number of contacts elicited, type of contact, number of contacts screened for TB symptoms, number of presumptive TB cases, number of presumptive TB cases evaluated for active TB, and number of presumptive TB cases with active TB. Types of contacts were household (child, spouse and other household member) and co-workers.

### Data management and analysis

The data were entered in EpiData 4.2.1.1 and analysed in Stata 16.0 (STATA, College Station, Texas, USA). Categorical variables were summarised as proportions while continuous variables were summarised as medians with the corresponding interquartile ranges (IQR). We performed binary logistic regression analysis to determine crude odds ratios (COR) for factors associated with having a contact investigation done and having a positive yield from the contact investigation. Thereafter, all factors with *p* < 0.2 were entered in a multivariate model and adjusted odds ratios (aOR) along with their respective 95% confidence intervals were determined. Therefore, we constructed two separate models; i. for factors associated with having a contact investigation and ii. For factors associated with having a positive contact tracing yield. Specifically, age, district, TB disease class and having a telephone contact were included in the model for factors associated with having a contact investigation. Type of index TB case, number of contacts screened for TB symptoms (per index case), number of presumptive TB cases (per index case) and number of presumptive TB cases evaluated for active TB (per index case) were included in the model for factors associated with having a positive contact tracing yield.

## Results

**Fig. 1:**
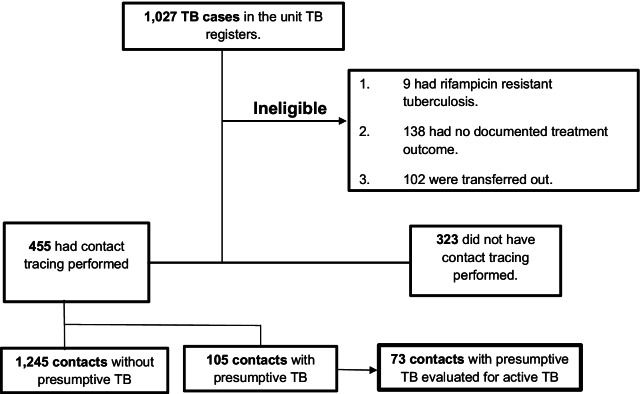
Study flow diagram

Data were extracted between February and March 2021. A total of 778 index cases were included after screening 1,027 cases. Among those excluded, 138 had no documented treatment outcome, 102 were transferred out while 9 had rifampicin resistant TB. Figure 1 shows the study flow diagram.

### Characteristics of index TB cases and contacts

Of 778 index cases, 455 (58.5%) had a contact investigation conducted overall. Among the 778 index cases, 689 (88.6%) were eligible for a contact investigation by national guidelines but contact tracing was performed for only 409 (59.4%) of these. The median (IQR) number of times an index case had a contact investigation done was 1 (1–2). A total of 1,350 contacts were elicited. Of these, 543 (40.2%) were household contacts, 4 (0.3%) were co-workers and the type of contact was unknown among 803 (59.5%). Among household contacts, there were 126 (23.2%) child contacts, 120 (22.1%) spouses and 292 (53.8%) other household contacts. Among the 1,350 contacts, 105 (7.8%) had presumptive TB. Of these, 73 (69.5%) were further evaluated for active TB and 29 contacts had active TB (14 of these had bacteriologically confirmed TB). The contact tracing yield for active TB was therefore 2.1% (29/1,350). Table 1 compares characteristics among cases that had a contact investigation with those who did not.

**Table 1:** Characteristics of index TB cases with and without a contact investigation

Characteristic	Contact investigation status		*p*-value
	Not done n (%)	Done n (%)	
Age (years) (n = 772)			0.037
< 15	21 (6.5)	21 (4.7)	
15–34	180 (56.1)	219 (48.6)	
34–60	117 (36.4)	200 (44.3)	
> 60	3 (0.9)	14 (2.4)	
Sex (n = 776)			
Male	214 (66.5)	312 (68.7)	0.506
Female	108 (33.5)	142 (31.3)	
Type of case (n = 763)			0.408
New	305 (95.9)	421 (94.6)	
Retreatment	13 (4.1)	24 (5.4)	
District (n = 774)			0.285
Kampala	237 (73.6)	331 (73.2)	
Wakiso	79 (24.5)	118 (26.1)	
Other	6 (1.9)	3 (0.7)	
Type of residence (n = 735)			0.754
Urban	288 (95.4)	415 (95.8)	
Rural	14 (4.6)	18 (4.2)	
TB disease class			< 0.001
PBC	161 (49.8)	290 (63.7)	
PCD	136 (42.1)	144 (31.6)	
Extrapulmonary TB	26 (8.0)	21 (4.6)	
HIV status			0.278
Positive	176 (54.5)	230 (50.5)	
Negative	147 (45.5)	225 (49.5)	
Case has phone contact			0.020
Yes	219 (67.8)	343 (75.4)	
No	104 (32.2)	112 (24.6)	

*TB* tuberculosis, *PBC* pulmonary bacteriologically confirmed TB, *PCD* pulmonary clinically diagnosed TB

### Factors associated with having a contact investigation conducted

Index cases were more likely to have a contact investigation if they had a telephone contact in the register (aOR 1.66, 95% CI 1.02–1.97, *p* = 0.036). Expectedly, pulmonary clinically diagnosed TB cases (aOR 0.60, 95% CI 0.44–0.83, *p* = 0.002) and extrapulmonary TB cases (aOR 0.48, 95% CI 0.26–0.89, *p* = 0.020) were less likely to have a contact investigation conducted. Table 2 shows factors associated with having a contact investigation.

**Table 2:** Factors associated with having a contact investigation conducted

Characteristic	Bivariate analysis		Multivariate analysis	
	OR (95% CI)	*p*-value	aOR (95% CI)	*p*-value
Age (years)				
< 15	1		1	
15–34	1.22 (0.64–2.30)	0.546	0.85 (0.44–1.66)	0.641
34–60	1.71 (0.90–3.26)	0.104	1.26 (0.64–2.46)	0.504
> 60	3.67 (0.89–15.06)	0.071	2.80 (0.65–12.03)	0.165
District				
Kampala	1		1	
Wakiso	1.07 (0.77–1.49)	0.690	1.10 (0.78–1.54)	0.598
Other	0.36 (0.09–1.45)	0.149	0.34 (0.08–1.45)	0.145
TB disease class				
PBC	1		1	
PCD	0.59 (0.43–0.80)	0.001	0.60 (0.44–0.83)	0.002
Extrapulmonary TB	0.45 (0.24–0.82)	0.010	0.48 (0.26–0.89)	0.020
Phone contact available				
No	1		1	
Yes	1.45 (1.06, 2.00)	0.020	1.42 (1.02–1.97)	0.036

*TB* tuberculosis, *PBC* pulmonary bacteriologically confirmed TB, *PCD* pulmonary clinically diagnosed TB

### Factors associated with having a positive contact tracing yield

The odds of a positive yield increased tenfold for every additional presumptive contact evaluated for active TB (aOR 10.1, 95% CI 2.95–34.66, *p* < 0.001). Also, retreatment index TB cases were more likely to yield a positive contact (aOR 7.69 95% CI 2.08–25.00, *p* = 0.002) than to new cases. Table 3 shows factors associated with a positive contact tracing yield among index cases.

**Table 3:** Factors associated with a positive contact tracing yield

Characteristic	Bivariate analysis		Multivariate analysis	
	cOR (95% CI)	*p*-value	aOR (95% CI)	*p*-value
Type of index TB case				
New case	1		1	
Retreatment case	6.25 (2.27–16.67)	< 0.001	7.69 (2.08–25.00)	0.002
Number of contacts screened for TB symptoms (per index case)				
0–1	1		1	
2–4	3.07 (0.84–11.18)	0.090	2.72 (0.56–13.10)	0.212
5–7	6.47 (1.62–25.86)	0.008	3.13 (0.54–18.27)	0.205
> 7	13.17 (3.09–56.18)	< 0.001	0.94 (0.09–10.0)	0.962
Number of presumptive TB cases (per index case)	5.62 (3.32–9.54)	< 0.001	1.12 (0.47–2.71)	0.790
Number of presumptive TB cases evaluated for active TB (per index case)	11.61 (5.62–24.01)	< 0.001	10.11 (2.95–34.66)	< 0.001

*cOR* crude odds ratio, *aOR* adjusted odds ratio, *TB* tuberculosis

## Discussion

In this study we determined factors associated with a positive contact tracing yield among index TB cases at an urban TB clinic in Kampala, Uganda. We found that the odds of a positive yield increased with the number of presumptive TB cases (per index case) evaluated for active TB. Furthermore, retreatment index TB cases were more likely to yield a positive TB case compared to new index TB cases. Unfortunately, more than 30% of contacts with presumptive TB were not evaluated for active TB. Further, 35% of index retreatment cases did not have a contact investigation conducted. This could explain why the yield of contact tracing in our study was very low (2%). It follows that contact tracing should aim to evaluate all contacts with presumptive TB and contacts of index retreatment cases for active TB to increase the yield of contact tracing. The yield of contact tracing in our study is within the estimate reported by other studies in Uganda (1.7–5.3%) [12–14]. It is also similar to the yield of 3% reported globally [8].

Retreatment cases are cases that have had a previous treatment for TB and include TB loss-to-follow up, relapse and treatment failure cases [16]. As such, they have a long duration of active TB before presenting for retreatment. In that time, they are likely to have transmitted the infection to more people than new cases would have [18]. Therefore, it is expected that retreatment cases have a higher yield of active TB among their contacts than new index cases. Moreover, 26% of retreatment cases in Uganda have drug resistant tuberculosis (DRTB) [19]. Therefore, prioritising contacts of retreatment cases may increase detection of community transmission of DRTB. These results further emphasise the need for optimising TB therapy the first time it is initiated to prevent treatment loss-to-follow up, failure or relapse which could increase TB transmission to contacts. Prospective studies could determine whether conducting contact tracing for all retreatment cases (regardless of smear status) increases yield compared to the current recommendations that focus on bacteriologically confirmed TB, children age fiver years and below and people with HIV.

We found that the odds of a positive yield increased with the number of contacts with presumptive TB (for each index case) that were evaluated for active TB. This highlights the need to complete the entire contact tracing cascade for each index case. The cascade of contact tracing begins with eliciting and tracing contacts for all eligible index cases. In our study, more than 40% of eligible index cases did not have a contact investigation conducted. This low fidelity to contact tracing is observed in urban TB clinics in Kampala where only 61% of eligible index cases are scheduled to have a home visit for contact tracing [13]. As shown in our study, having a telephone contact of the index case increased the likelihood of having a contact investigation performed. Index cases can easily be contacted by health workers on the phone to elicit contacts and schedule home visits [20]. It is unclear why many contacts with presumptive TB in our study were not evaluated for active TB. However, in this setting, high travel costs to the health facility are identified by contacts as a barrier to TB evaluation [21]. Further, urban dwellers are very mobile, and this could pose challenge in following them up for TB evaluation. Nonetheless, TB cases in Kampala are less likely to be mobile compared to controls [22]. Future studies need to further explore barriers to completion of the contact screening cascade. A key element of the contact tracing cascade is identifying people eligible for tuberculosis preventive therapy (TPT) and initiating them on TPT to prevent future TB reactivation. Therefore, in evaluating the completeness of contact tracing cascade, programs and future studies should consider reporting rates of TPT initiation and completion among eligible contacts of index cases. Unfortunately, these data were not collected in the primary study.

Studies that have evaluated factors associated with positive contact tracing yield have reported varied results. Similar to our finding, Baliashvili and colleagues [18] found that contacts of retreatment index TB cases had higher prevalence of latent TB infection than new cases in Georgia. In Uganda, characteristics of adult index TB cases were not associated with incident active TB among child contacts [23]. However, only sex, sputum smear grade and extent of disease on chest x-ray were evaluated. In that study, sleeping in the same bedroom with the index case was associated with incident TB in the contacts. In South Africa, only self-reported cough among household contacts was associated with active TB [24]. Similar to our study, number of contacts per index case were not associated with a positive yield in these studies [23, 24].

The study has some limitations. The use of TB symptoms as an initial screening tool among contacts is limited by subjectivity and low sensitivity [25]. This could under-estimate the yield of contact tracing. However, this is the cheapest and readily available mechanism of TB screening in resource-limited settings. Another limitation is the unavailability of data on characteristics of the contacts (other than their relationship to the index case). This prevented us from evaluating characteristics such as age, immune status, and lifestyle of contacts that would influence the risk of acquiring TB from an index case. These data are not routinely recorded in the registers. Nevertheless, our study identifies a further sub-group of people with TB (retreatment cases) that would benefit from contact tracing investigations to maximise the yield of this endeavour. Another limitation is that index cases might have enumerated few contacts due to perceived stigma associated with having TB. Additionally, the type of contact was unknown in most cases. This might have affected us in determining which type of contacts are likely to yield a positive screen.

## Conclusion

The yield of active TB cases during contact tracing was low at an urban TB clinic in Uganda but was comparable to global estimates. The yield was higher in retreatment index TB cases compared to new cases. Also, the yield increased with the number of contacts with presumptive TB that were evaluated for active TB. TB contact tracing should aim to evaluate all presumptive TB contacts and contacts of retreatment cases to maximise the yield of contact tracing.

## Supplementary Information

**Additional file 1:** Intensified Case Finding Form.

## Data Availability

The datasets used and/or analysed during the current study available from the corresponding author on reasonable request.

## Abbreviations

TB: Tuberculosis
DRTB: Drug resistant tuberculosis
KHC: Kisenyi Health Center
cOD: Crude odds ratio
aOR: Adjusted odds ratio
PBC: Pulmonary bacteriologically confirmed
PCD: Pulmonary clinically diagnosed
IQR: Interquartile range
WHO: World Health Organisation
