# Supplementary material for: Tuberculosis contact tracing yield and associated factors in Uganda
Source: BMC Pulm Med. 2022 Feb 16;22:64. doi: 10.1186/s12890-022-01860-z (PMC8848908; doi:10.1186/s12890-022-01860-z)
Supplement: Supplementary file 1 — Additional file 1: Intensified Case Finding Form. [file 12890_2022_1860_MOESM1_ESM.docx]

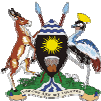
**Appendix: Intensified case finding form**

Ministry of Health

*Use the form to suspect:*

*TB in People living with HIV, contacts of smear positive cases and in HIV care settings*

***This form should be administered by either a health care provider or lay provider at the health facility (The form should not be self- administered)***

Date of TB Assessment: Name of district:

Name of Health facility: Location : ( e.g. OPD, HIV Clinic etc.) Name of Individual: Age: Sex:

District of Residence: Sub county: Village/LCI/Zone:

1. Has the patient been coughing for 2 weeks or more? **Yes** □ **No**□
2. Has the patient coughed up sputum stained with blood? **Yes**□ **No**□
3. Has the patient had persistent fevers for 3 weeks or more? **Yes** □ **No**□
4. Has the patient had noticeable weight loss (more than 3 kg) in the last one month?

**Yes** □ **No**□

1. Has the patient had night sweats for 3 weeks or more? **Yes** □ **No**□ **Guide for Actions to take**

- If **yes to question 1 or 2;** request for sputum test and refer to clinician for further investigations. ***Direct the patient to a designated area for people with chronic cough.***
- **If no to question 1 and 2 and yes to any other question;** refer to clinician for further investigations
- **If no to all questions:** repeat TB Assessment at subsequent visits

**Record of Information at Health facility level**

- 1. *If you are in a clinic attending to patients enrolled in HIV care record this information on the comprehensive ART card; this information should then be transferred to the Pre ART or ART register.*
  2. *If you are in any HIV care setting (not attending to patients enrolled in HIV care e.g. OPD) and the patient is found to be a TB suspect record this information in a TB suspect register.*
